# Draft genome assemblies of the avian louse *Brueelia nebulosa* and its associates using long-read sequencing from an individual specimen

**DOI:** 10.1093/g3journal/jkad030

**Published:** 2023-02-03

**Authors:** Andrew D Sweet, Daniel R Browne, Alvaro G Hernandez, Kevin P Johnson, Stephen L Cameron

**Affiliations:** Department of Biological Sciences, Arkansas State University, 2713 Pawnee Street, Jonesboro, AR 72401, USA; Pacific Biosciences, 1305 O’Brien Drive, Menlo Park, CA 94025, USA; Roy J. Carver Biotechnology Center, University of Illinois at Urbana-Champaign, Urbana, IL 61801, USA; Illinois Natural History Survey, Prairie Research Institute, University of Illinois at Urbana-Champaign, Champaign, IL 61820, USA; Department of Entomology, Purdue University, West Lafayette, IN 47907, USA

**Keywords:** ectoparasite, *Sturnus vulgaris*, PacBio, TELL-Seq, historical effective population size, mitogenome, endosymbiont

## Abstract

Sequencing high molecular weight (HMW) DNA with long-read and linked-read technologies has promoted a major increase in more complete genome sequences for nonmodel organisms. Sequencing approaches that rely on HMW DNA have been limited to larger organisms or pools of multiple individuals, but recent advances have allowed for sequencing from individuals of small-bodied organisms. Here, we use HMW DNA sequencing with PacBio long reads and TELL-Seq linked reads to assemble and annotate the genome from a single individual feather louse (*Brueelia nebulosa*) from a European Starling (*Sturnus vulgaris*). We assembled a genome with a relatively high scaffold N50 (637 kb) and with BUSCO scores (96.1%) comparable to louse genomes assembled from pooled individuals. We annotated a number of genes (10,938) similar to the human louse (*Pediculus humanus*) genome. Additionally, calling phased variants revealed that the *Brueelia* genome is more heterozygous (∼1%) then expected for a highly obligate and dispersal-limited parasite. We also assembled and annotated the mitochondrial genome and primary endosymbiont (*Sodalis*) genome from the individual louse, which showed evidence for heteroplasmy in the mitogenome and a reduced genome size in the endosymbiont compared to its free-living relative. Our study is a valuable demonstration of the capability to obtain high-quality genomes from individual small, nonmodel organisms. Applying this approach to other organisms could greatly increase our understanding of the diversity and evolution of individual genomes.

## Introduction

Long-read sequencing technology, such as those from Pacific Biosciences (PacBio) and Oxford Nanopore Technologies (ONT), has led to a significant advance in assembling high-quality genomes (Burgess *et al*. 2018; Pollard *et al*. 2018; Mantere *et al*. 2019; Amarasinghe *et al*. 2020; Logsdon *et al*. 2020). This is particularly true for nonmodel organisms, which usually do not have highly inbred or clonal lineages that can help improve genomic assemblies with short-read data, or are from less common species that cannot be pooled to obtain high amounts of genomic material (Larsen *et al*. 2014; da Fonseca *et al*. 2016; Guiglielmoni *et al*. 2021). However, these approaches have been unrealistic for smaller organisms due to low yields of high molecular weight (HMW) DNA or issues with specimen storage (Post *et al*. 1993; Schalamun *et al*. 2019; Blom 2021; Dahn *et al*. 2022; Trigodet *et al*. 2022). Nevertheless, recent advances in library preparation have helped overcome some of the previous limitations of long-read sequencing for smaller organisms. For example, PacBio released low and ultralow library protocols, which allow for lower input (minimum 100 and 5 ng, respectively) of HMW DNA in preparation for sequencing with SMRT technology (Duncan *et al*. 2019; Kingan *et al*. 2019; Schneider *et al*. 2021). This advancement has enabled high-quality long-read sequencing from individuals of small organisms that were stored in a variety of conditions, including sequences of their mitochondrial genomes (mitogenomes) and bacterial endosymbionts (Kumar and Blaxter 2011; Meng *et al*. 2019).

Parasitic lice (Insecta: Psocodea) are small (usually ∼1 mm in length) insects that parasitize mammals and birds and are one group of nonmodel organisms that possess limitations for obtaining high-quality genome assemblies due to their small size and challenges obtaining large numbers of individuals from wild populations (i.e. for pooling; Marshall 1981; Sychra *et al*. 2011). Although there are ∼5,000 described species of parasitic lice (Durden and Musser 1994; Price *et al*. 2003), there are draft genomes available from only 2 species: the human body louse (*Pediculus humanus huanus* L.; Kirkness *et al*. 2010) and the slender pigeon louse (*Columbicola columbae* L.; Baldwin-Brown *et al*. 2021). Both of these genomes were generated from hundreds or thousands of pooled individuals. Here, we sequenced the genome of an individual feather louse in the genus *Brueelia* from a European Starling (*Sturnus vulgaris* L.) using a combination of HiFi reads (highly accurate long reads) from PacBio and TELL-Seq (barcode-linked short reads) from Universal Sequencing (UST). We also tested the ability to obtain long-read sequence data from specimens stored in different conditions. We report the draft assembly and initial annotation of the assembled scaffolds, called and phased variants, and used the variant information to calculate heterozygosity and reconstruct the historical effective population size of the louse. We also assembled and annotated the genome from the primary endosymbiont and the mitogenome. To our knowledge, this is the first use of HiFi reads and TELL-Seq sequencing technology applied to parasitic lice and a substantial step forward in elucidating genomic information from an individual louse, paving the way for larger scale studies of populations of nonmodel organisms at individual resolution.

## Methods

### Sample acquisition

We collected samples of lice from a recently deceased European Starling (*S. vulgaris*) recovered in Lafayette, IN, USA and a live Turkey Vulture (*Cathartes aura* L.) from the Wildcat Wildlife Center (Delphi, IN, USA). We collected lice from *S. vulgaris* using ethyl acetate fumigation, immediately placed them in 95% ethanol, and stored them either in a −80°C freezer within 24 h of collection or at room temperature. Lice from *C. aura* were collected live, placed in a vial, and immediately frozen at −20°C and then −80°C. We identified the lice to genus using Price *et al*. (2003): *Brueelia* from *S. vulgaris* and *Colpocephalum* from *C. aura*.

### Extractions and sequencing

We used several specimens and extraction methods to test the effectiveness of different specimen storage and extraction protocols for obtaining HMW DNA from lice. We used specimens of lice stored in 95% ethanol at room temperature for ∼4 months, stored in ethanol at −80°C (both from *S. vulgaris*), and fresh specimens (not in ethanol) stored at −80°C (from *C. aura*). We then extracted HMW DNA from single lice (i.e. not pooled samples) in each of the 3 storage categories, 2 lice from each category. Before extractions, we photographed each louse as a voucher using a Leica M165 C in the Purdue Entomological Research Collection (PERC) at Purdue University, West Lafayette, IN, USA. All extractions were done at the Roy J. Carver Biotechnology Center at the University of Illinois (Champaign, IL, USA). Briefly, HMW DNA extraction was performed with the MagAttract kit (Qiagen, Valencia, CA, USA) with a slightly modified protocol. Specimens were transferred to a 1.5-ml tube, 20 μl of lysis buffer were added and samples were ground with a plastic pestle. The tube was incubated at 25°C for 1 h. After incubation, 15 μl of magnetic beads and 140 μl of buffer MB were added and the tube was rotated for 15 min as described in the manufacturer's protocol. After bead washing, the DNA was eluted twice with 12.5 μl of AE buffer each time, at 40°C for 10 min. The DNA was quantitated with a Qubit High Sensitivity kit (ThermoFisher, Waltham, MA, USA) and the integrity was evaluated in a Fragment Analyzer (Agilent Technologies, Santa Clara, CA, USA).

We sequenced the sample with the highest level of HMW DNA (female *Brueelia* stored in ethanol at −80°C) using a Sequel II system (PacBio, Menlo Park, CA, USA), and TELL-Seq linked reads (UST, Canton, MA, USA) on a NovaSeq 6000 system (Illumina, San Diego, CA, USA). All sequencing was carried out at the Roy J. Carver Biotechnology Center at the University of Illinois. The HMW DNA was sheared with a Megaruptor 3 (Diagenode, Denville, NJ, USA) to an average fragment length of 10 kb. Library construction was performed from 5 ng of sheared DNA with an UltraLow DNA Input kit (PacBio, Menlo Park, CA, USA), which involves ligation of adaptors to the sheared DNA and PCR amplification under conditions that favor both AT rich as well as well-balanced and GC rich portions of the genome, followed by library preparation with an SMRTBell Express Template Prep 2.0 kit (PacBio, Menlo Park, CA, USA). Sequencing was performed on the Sequel II system using an SMRT Cell 8 M (PacBio, Menlo Park, CA, USA) with a 30-h movie time. The HiFi reads files [in BAM and FASTQ format) were generated with SMRT Link 8.0 (PacBio, Menlo Park, CA, USA)] using the following parameters: minimum length of 1,000 bases, minimum number of passes of 3, and minimum predicted consensus accuracy of 99%.

Linked-read TELL-Seq libraries (UST, Canton, MA, USA) were prepared from the same HMW DNA that was used to make the PacBio library. The TELL-Seq library was quantitated with a Qubit, run on a Fragment Analyzer, and sequenced with a NovaSeq 6000 SP Reagent Kit v1 (300 cycles) lane (Illumina, San Diego, CA, USA), yielding 2 × 150 bp paired-end short reads.

### Genome size estimation

We estimated the genome size and heterozygosity of the *Brueelia* genome using reads from the TELL-Seq sequencing. We generated a count of k-mers in jellyfish (Marcais and Kingsford 2011) with a k-mer length of 21 and used this file to estimate genome statistics in GenomeScope2 (Ranallo-Benavidez *et al*. 2020).

### Louse genome assembly

Before assembling the HiFi reads, we trimmed adapters and removed PCR duplicates using 2 utilities from SMRT Link 8.0: lima v1.11.0 to trim PCR adapters and pbmarkdup v1.0.0 to mark PCR duplicates (PacBio, Menlo Park, CA, USA). We also identified possible contaminant reads by mapping against the genomes of possible contaminant organisms using pbmm2 v1.4.0 (PacBio, Menlo Park, CA, USA) or using Kraken2 v.2.1.1 (Wood *et al*. 2019) against the Greengenes (2019) and Fungal genomes (2019) databases on the Galaxy web platform (Afgan *et al*. 2016). For pbmm2, we mapped the reads against the NCBI human RefSeq genome (Build 38, patch 13), NCBI RefSeq bacterial reference genomes, and the *S. vulgaris* genome (the host; GCA_001447265.1). However, none of the mappings or Kraken2 searches identified more than 0.86% of reads and removing these reads resulted in less complete genome assemblies (Supplementary Table 1). These reads may have been from highly conserved regions of the genome, with similarity broadly across species, rather than true contaminants. Therefore, we proceeded with the assembly without removing possible read contaminants. We trimmed the raw TELL-Seq reads with TellRead v.1.0.2 (UST, Canton, MA, USA) and reformatted the trimmed reads for scaffolding using scripts from UST (https://www.universalsequencing.com/analysis-tools).

We used a combination of several approaches to assemble the *Brueelia* genome. First, we de novo assembled the trimmed HiFi reads using IPA v1.0.5 (PacBio, Menlo Park, CA, USA), HiCanu v.2.1.1 (Nurk *et al*. 2020), Hifiasm v.0.13 (Chen *et al*. 2020), and Flye v.2.8.1 (Kolmogorov *et al*. 2019). We used an estimated HiFi read error rate of 0.001 for Flye. For each assembly, we calculated average coverage by mapping reads using pbmm2, calculated assembly statistics using QUAST v.5.0.2 (Gurevich *et al*. 2013), and estimated assembly completeness using BUSCO v.4.0.6 with the insecta_odb10 database (Simão *et al*. 2015). We then combined each of these assemblies in Flye using the –subassemblies command, and once again estimated depth, assembly statistics, and completeness.

We used BLAST searches to identify possible contaminants or other elements not part of the nuclear genome among the assembled contigs. We ran BLAST searches against the NCBI RefSeq Genome database (Altschul *et al*. 1990) and assessed the taxonomy of the top 10 BLAST hits using the R package primerTree (Hester 2020). We also searched for a potential mitogenome sequence by running a BLAST search against a published sequence of the cytochrome oxidase subunit I (*cox1*) gene from *Boeckella antiqua* Ansari, 1956 (NCBI accession # FJ71222). Based on these searches, we removed 4 contigs that returned high bit scores and low *e*-values: one from a likely bacterial contaminant (*Cutibacterium acnes*), one that is likely the mitogenome and 2 that had high similarities to *Sodalis*, the primary bacterial endosymbiont of many species of lice (Boyd and Reed 2012; Supplementary Table 2 and Supplementary Fig. 1).

Second, we used ARCS v.1.1.1 (Yeo *et al*. 2018) with the TELL-Seq linked reads to assemble scaffolds from the trimmed contigs. We used the default settings in the arcs-tigmint pipeline scripts, which uses a combination of ARCS and LINKS (Warren *et al*. 2015) to scaffold contigs using linked read information. After scaffolding, we once again estimated depth, statistics, and completeness as described above. All assemblies were run on the Bell Cluster maintained by Information Technology at Purdue (Two Rome 2.0 GHz processors, 128 cores, 256 GB memory).

### Annotation

We identified repeat regions of the assembled scaffolds using RepeatModeler v.1.0.9 (https://www.repeatmasker.org/) and RepeatMasker v.4.0.7 (https://www.repeatmasker.org/RepeatModeler/). We then annotated the scaffolds using MAKER v.2.31.10 (Holt and Yandell 2011). First, we trained AUGUSTUS (Stanke *et al*. 2006) gene prediction models in BUSCO using the *insect_obd10* single-copy ortholog set. We then ran gene predictions in MAKER using AUGUSTUS and protein sequences from the SwissProt database (release 2021_3) and 5 published genomes of related insect taxa: *Acyrthosiphon pisum* Harris, 1776 (pea aphid; GCD_005508785.1), *Bemisia tabaci* Gennadius, 1889 (whitefly; GCF_001854935.1), *C. columbae* (slender pigeon louse; GCA_016920875.1), *P. humanus* (human body louse; GCA_000006295.1), and *Drosophila melanogaster* Meigen, 1830 (fruit fly; Release 6 plus ISO1_MT). After running MAKER, we removed any gene predictions with Annotation Edit Distance (AED) scores >0.5 using the quality_filter.pl script for GFF files (from https://github.com/mscampbell/Genome_annotation) and a custom Python script for FASTA files (available at https://github.com/adsweet/louse_genomes). We then assigned functional annotations to the predicted genes using Pfam in InterProScan v.5.36–75.0 (Zdobnov and Apweiler 2001) and *blastp* against the Swiss-Prot database (downloaded on November 7, 2022), both on the Galaxy web platform. Finally, we compared our predicted genes in *Brueelia* with single-copy orthologous genes in 2 louse genomes (*C. columbae* and *P. humanus*) and *D. melanogaster* using OrthoVenn2 (Xu *et al*. 2019) with an *E*-value of 1e-5 and Inflation value of 1.5.

### Phasing and variant calling

To estimate heterozygous variants across the *Brueelia* genome, we called and phased variants using the assembled scaffolds (contigs from combined Flye subassemblies of HiFi reads, scaffolded with TELL-Seq reads and ARCS). First, we mapped our HiFi reads to the scaffolds using pbmm2. Next, we used HaplotypeCaller in GATK to call variants using an aggressive PCR indel model (Van der Auwera and O’Connor 2020). We then filtered variants using VariantFiltration (QD < 2.0, FS > 60.0, MQ < 40.0, MQRankSum < −12.5, ReadPosRankSum < −8.0) and removed filtered sites with SelectVariants. Finally, we phased the filtered variants using WhatsHap v.1.0 (Martin *et al*. 2016). We summarized variants in 1-kb windows across the scaffolds using vcftools v.0.1.16 (Danecek *et al*. 2011).

### Population demographic history

We used our HiFi reads mapped against the scaffolds to estimate the demographic history of our *Brueelia* sample with the Pairwise Sequentially Markovian Coalescent (PSMC) model, which models changes in effective population size (*N_e_*) through time from individual diploid genome sequences (Li and Durbin 2011). We used SAMtools v.1.8 (Li *et al*. 2009) and BCFtools v.1.8 (Danecek *et al*. 2021) to convert our mapped reads for input into PSMC (https://github.com/lh3/psmc). We ran 100 bootstrap replicates of PSMC with 64 atomic time intervals (-p 28*2 + 2 + 6) and default values of -t and -r. These parameters were chosen to ensure that at least 10 recombinations occurred in each parameter interval (Li and Durbin 2011). We plotted *N_e_* through time based on a generation time of 1/12 (0.08) of a year and a mutation rate of 8.4 × 10^−9^ based on estimates in *Drosophila* (Haag-Liautard *et al*. 2007).

### Mitogenome assembly and annotation

The BLAST search with our subassemblies from Flye against a published sequence of *cox1* from *B. antiqua* identified a 14,409 bp contig with a high bit-score (352.94) and low *e*-value (2.33e−96). We removed this contig for downstream scaffolding, but ran separate analysis to test whether this contig is the complete mitogenome from our *Brueelia* sample. We annotated the contig with the MITOS2 web server using the Metazoan RefSeq reference set (Donath *et al*. 2019). We then manually curated the annotations by identifying Open Reading Frames for protein coding genes and comparing the annotations to a previously assembled mitogenome from *B. antiqua* (Sweet *et al*. 2022). We then tested for circularity of the contig using the paired-end reads from TELL-Seq in AWA v.1.0, which maps paired reads against the merged 5′ and 3′ ends of a contig to test for circularity using depth and mapping scores (Machado *et al*. 2018). Finally, we used the annotated *cox1* gene in a BLAST search against the NCBI nucleotide database to aid in the species-level identification of our sample of *Brueelia*. Several studies have focused on the phylogeny and taxonomy of *Brueelia* (Bush *et al*. 2016; Sweet *et al*. 2018), so there is considerable mitochondrial data from Sanger sequencing available on NCBI (1,213 nucleotide sequences, as of February 21, 2022).

### Endosymbiont assembly and annotation

To confirm our assembly of a *Sodalis* endosymbiont, we ran blastn searches against the subassembly contigs from Flye. We used nucleotide sequences of the *aroK*, *ftsA*, *mraY*, and *secY* genes from the genome of *Sodalis praecaptivus* HS1 strain (NZ_CP006569) as queries. All searches identified the same 1.8 Mbp contig. This contig was also one we had identified in our decontamination steps using BLAST searches against the NCBI RefSeq database. We annotated this contig using Prokka v.1.14.6 on the Galaxy web platform with –genus set to *Sodalis* and a minimum *e*-value cutoff of 1e−6 (Seemann 2014). We also tested for circularity of the contig using the TELL-Seq reads in AWA. Finally, we compared synteny blocks between our prospective *Sodalis* genome and the *S. praecaptivus* HS1 genome using the “loose” parameter in Sibelia v.3.0.7 (Minkin *et al*. 2013). We visualized the resulting synteny using Circos (Krzywinski *et al*. 2009).

## Results and discussion

### Ideal specimen storage and extraction protocols for HMW DNA in lice

**Fig. 1.**
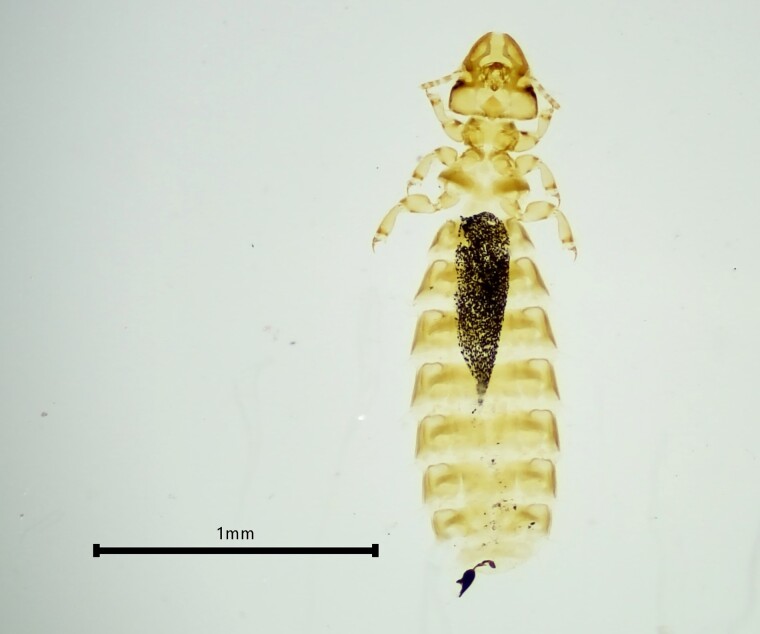
Photograph of a *Brueelia* louse collected from a European Starling (*Sturnus vulgaris*). The dark area is remaining gut content (likely feathers) after clearing and mounting in Canada balsam.

The *Brueelia* louse from *Sturnus vulgaris* (Fig. 1) stored in 95% ethanol at −80°C yielded enough HMW DNA (∼12 ng) for the PacBio UltraLow Input protocol (about 10 ng needed). The samples stored in ethanol at room temperature and frozen after being collected live from a Turkey Vulture (*C. aura*) did not yield any readable HWM DNA for long-read sequencing. These results suggest it is best to store specimens in ultracold temperatures as soon as possible to ensure the preservation of HMW DNA. Cold temperatures even seem to preserve material effectively in ethanol, which can result in a higher degradation of HMW DNA compared to other storage solutions (i.e. in ethanol at room temperature) or immediately freezing a live specimen (Oosting *et al*. 2020). In our cases, the lice from Turkey Vultures were likely not stored in ultracold conditions soon enough; the specimens were stored for 2–3 days at room temperature or −20°C before being transferred to a −80°C freezer. Future work could conduct a more extensive comparison using many replicates of storage treatments (we only had 2 replicates per storage condition), but our results suggest that specimens stored at room temperature for more than a few days are less reliable for HMW sequencing efforts. Nevertheless, lice are often collected directly into 95% ethanol and stored in ultracold conditions soon thereafter, which suggests there are many samples in existing research collections which could be useful for long-read sequencing approaches.

### Sequencing, assembly, and annotation of the *Brueelia* genome

**Fig. 2.**
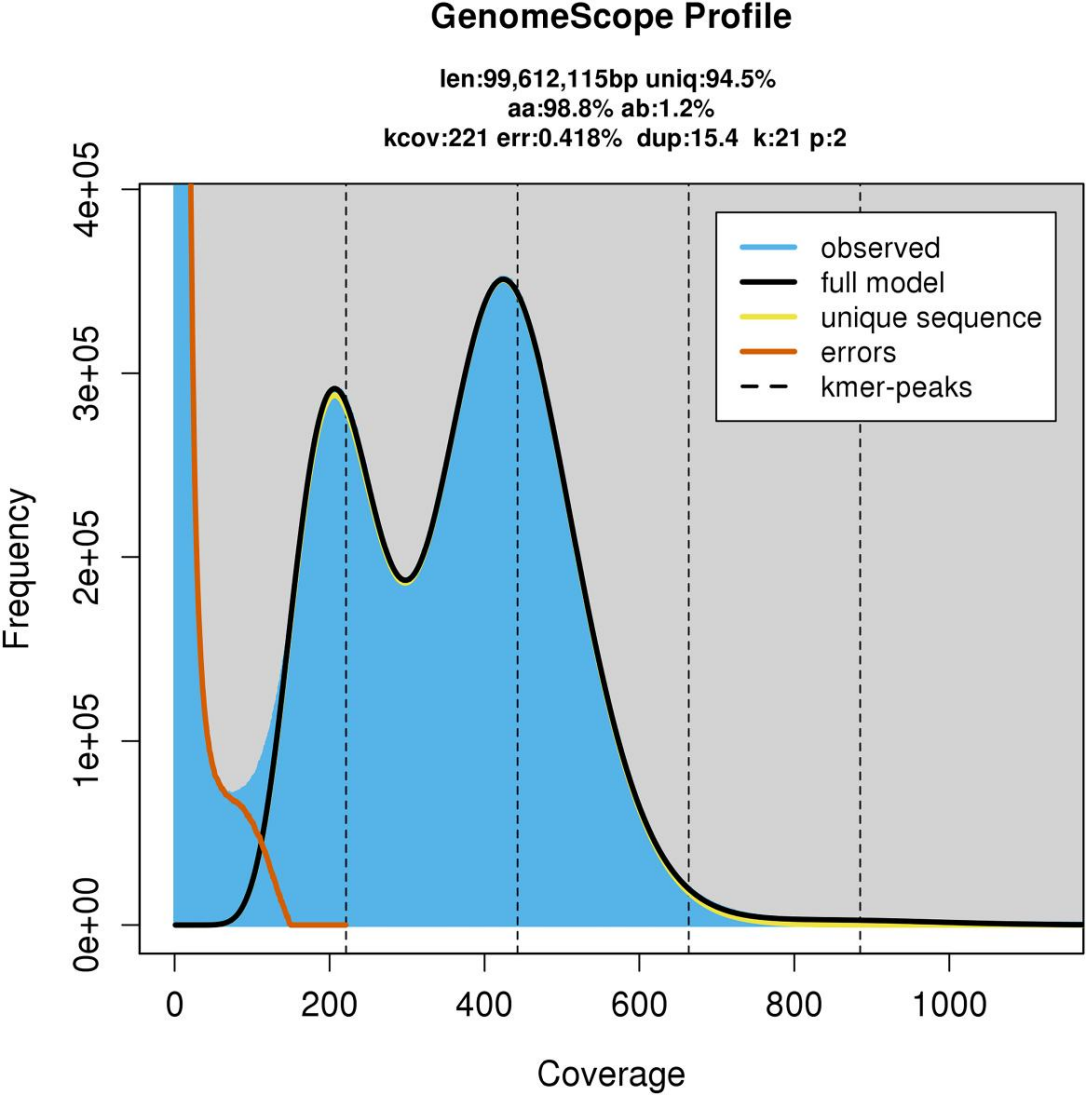
Distribution of k-mer frequencies in *Brueelia nebulosa* from GenomeScope2 using 150 bp Illumina reads and a k-mer size of 21. The profile includes estimates of total genome length (len), rate of heterozygosity (ab), and mean k-mer coverage for heterozygous bases (kcov). Double peaks in the observed distribution of k-mers indicate a heterozygous diploid genome.

Sequencing on the PacBio Sequel II system and SMRT Cell 8 M generated ∼20.1 Gbp of HiFi reads data. These consisted of 2,163,626 HiFi reads with an average length of 9,289 bp and maximum length of 27,483 bp. After removing adapters and PCR duplicates, 2,081,199 HiFi reads remained for downstream analysis. Sequencing of the TELL-Seq library generated 245,526,001 raw paired reads and 241,120,541 paired reads after filtering. Based on the distribution of k-mer counts from filtered reads, GenomeScope estimated a haploid genome size of 99.5 Mbp and 1.2% heterozygosity (Fig. 2). This genome size would be smaller than, but consistent with, the genome sizes of other species of lice, including *C. columbae* (∼208 Mbp) and *P. humanus* (∼110 Mbp; Kirkness *et al*. 2010; Baldwin-Brown *et al*. 2021).

Assembly of the HiFi reads with IPA, HiCanu, Hifiasm, Flye, and a combination of subassemblies with Flye generated high coverage assemblies (all average >79X), but with variable statistics of completeness (Tables 1 and 2). The IPA assembly had the fewest number of contigs (491), and highest contig N50 (∼293 kbp), but the smallest length (∼98 Mbp) and lowest BUSCO score (90.4% complete, single-copy orthologs). The Flye assembly had a higher BUSCO score (92.9%) than the IPA assembly, but a lower contig N50 (∼95 kbp). HiCanu and Hifiasm assemblies also had higher BUSCO scores (93.1% and 93.2%, respectively), but with lower contig N50s (∼74 and ∼43 kbp, respectively) and nearly double the total length (∼223 and ∼234 Mbp, respectively). The combined subassemblies with Flye seemed to combine the strengths of each assembly, with an N50 comparable to IPA (∼281 kbp), high BUSCO score (96.4%), and lower total length (∼116 Mbp). It is likely HiCanu and Hifiasm assembled separate haplotypes, which would explain the difference in total length compared to the other assemblies. Notably, IPA and Flye are haplotype-aware or phased assemblers, which suggests high heterozygosity (as indicated by the GenomeScope analysis) likely compounds the issue of assembling separate haplotypes in HiCanu and Hifiasm. High heterozygosity can also result in a more fragmented assembly, which are reflected in the relatively lower N50s and number of contigs in our assemblies (Ruan and Li 2020; Guiglielmoni *et al*. 2021). Nevertheless, the N50s and BUSCO scores of our assemblies are comparable to or exceed those of previous assemblies from pooled samples of lice.

**Table 1.** Statistics for the assembly of an individual *Brueelia nebulosa* louse using PacBio and TELL-seq data.

Assembly software	Average coverage	Number of contigs	Total length	Scaffold N50	GC%	BUSCO complete (%)	BUSCO after contaminant removal (%)
IPA	184.3	491	97,886,543	293,403	38.1	90.4	34.2
HiCanu	86	5612	222,948,165	74,003	38.4	94.1	93.1
Hifiasm	79.8	6750	234,315,027	42,931	38.4	93.2	93.2
Flye	114.8	2519	163,962,268	94,640	38.1	92.9	92.2
Flye + subassemblies	164.9	2205	115,935,770	281,302	38.11	96.4	78.8
Flye + ARCS	164.3	1675	113,962,985	636,874	37.9	96.1	—

**Table 2.** Percentage of BUSCO groups from the Insecta lineage (out of 1,367) identified or missing from different assemblies of *Brueelia nebulosa* using PaBio and TELL-seq data.

Assembly software	Complete	Complete and single copy	Complete and duplicated	Fragmented	Missing
IPA	90.4	85.6	4.8	0.8	8.8
HiCanu	94.1	16.2	77.9	1.0	4.9
Hifiasm	93.2	21.2	72.0	1.4	5.4
Flye	92.9	39.2	53.7	1.2	5.9
Flye + subassemblies	96.4	89.1	7.3	0.6	3.0
Flye + ARCS	96.1	89.4	6.7	0.9	3.0

Scaffolding the decontaminated contigs from the Flye-combined subassemblies with TELL-Seq linked reads helped to improve the assembly (Table 1). The total length and BUSCO score of the scaffolds from ARCS were similar to those from the Flye subassemblies (∼114 Mbp length, 96.1% BUSCO score; Table 1), but the ARCS assembly had a considerably larger N50 (∼637 kbp) and nearly half the number of scaffolds (1,684). This indicates that HiFi reads from the UltraLow Input kit are able to assemble most of the nuclear genome, but scaffolding with linked reads-like TELL-Seq can greatly decrease the fragmentation of the assembly and get closer to a chromosome-level, telomere-to-telomere assembly for an individual louse. Therefore, we used the scaffolds produced by ARCS for downstream annotation and variant analysis. The scaffolded assembly also indicates the total size of the nuclear genome is ∼114 Mbp. Again, this is consistent with the genome sizes of the pigeon wing louse *Columbicola* (∼208 Mbp) and the human body louse *Pediculus humanus* (∼110 Mbp). The GC content of the ARCS scaffolds was similar to the other assemblies (37.9%), which is consistent with the GC content of most other insect nuclear genomes (Li *et al*. 2019).

### Annotation

RepeatMasker identified 17.2 Mbp (15.05%) of repetitive content in the ARCS scaffold assembly. This included 2.8 Mbp of DNA transposons, 539.8 kbp of LINEs, 1.7 Mbp of simple repeats, and 73.2 kbp of LTR transposons (Table 3). RepeatMasker did not identify any SINEs. Most of the remaining repetitive content was unclassified (11.5 Mbp). This level of repetitive content is higher than in *Columbicola* (9.7%) and *Pediculus* (7%) (Kirkness *et al*. 2010; Baldwin-Brown *et al*. 2021).

**Table 3.** Annotation statistics for the scaffolds of *Brueelia nebulosa* assembled using PacBio reads in Flye and scaffolded using linked TELL-Seq reads in ARCS.

Number of genes	10,938
Number of genes with AED <0.5	10,587
Mean gene length	3,581 bp
Number of exons	69,753
Mean exon length	263 bp
LINEs	0.47%
LTR elements	0.06%
DNA elements	2.45%
Total interspersed repeats	13.05%
Simple repeats	1.46%

**Fig. 3.**
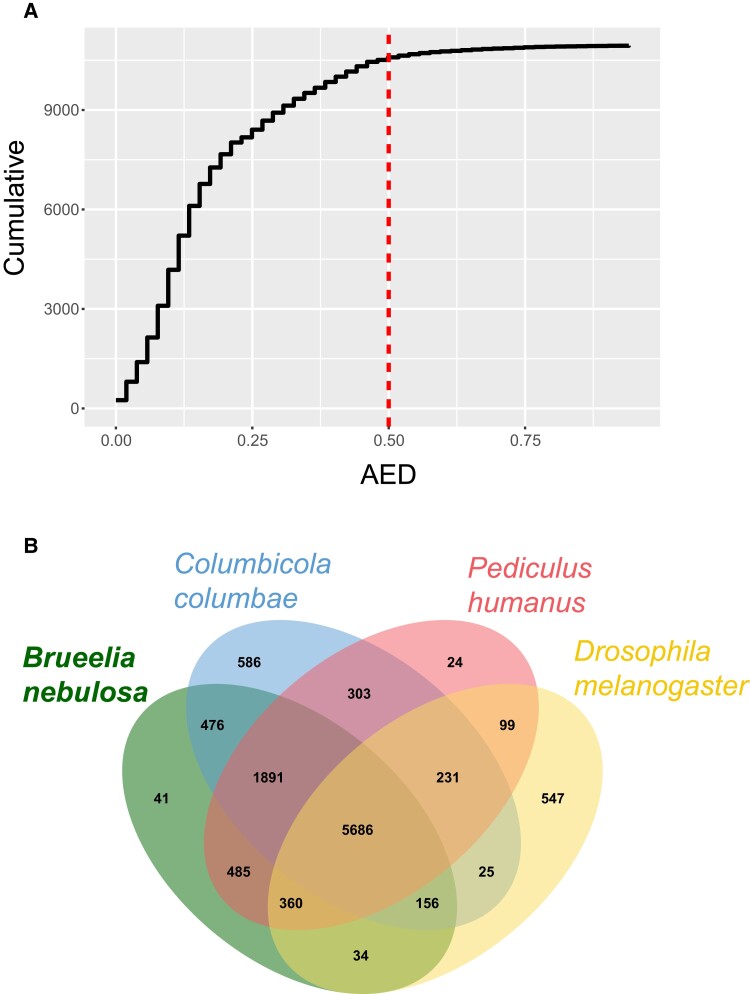
a) the cumulative AED from genes identified with the MAKER pipeline on assembled scaffolds of *Brueelia nebulosa*. Genes with AED scores >0.50 are considered low quality. b) Venn diagram of orthologous gene clusters from the genomes of *B. nebulosa*, 2 other species of parasitic lice (*Columbicola columbae* and *Pediculus humanus*), and *Drosophila melanogaster*.

Our annotation with the MAKER pipeline identified 10,938 genes from the scaffolded assembly (Table 3). We only removed 351 (3.2%) of these genes due to high AED scores (>0.5). A total of 249 of the genes (2.3%) had AED scores of 0 (Fig. 3a). Of the 10,587 filtered genes, we were able to assign functional annotations to 9,926 of them (93.8%). The number of transcripts is likely considerably lower than the actual number in the nuclear genome, given the number of annotated genes in the *Columbicola* genome (>13,000) (Baldwin-Brown *et al*. 2021). This is likely due to the lack of transcriptomic data used in our assembly (Trapnell *et al*. 2010). It is currently not feasible to easily obtain transcriptome data from an individual louse (pers. obs.). However, our result is likely a good draft annotation, given the low AED scores and percentage of transcripts assigned a functional annotation. Our gene number is also similar to the *Pediculus* genome (10,993; Kirkness *et al*. 2010). In addition, comparisons among the genes of our *Brueelia* genome, the *Pediculus*, *Columbicola*, and *D. melanogaster* with OrthoVenn indicated a high amount of shared orthologous gene clusters (Fig. 3b). A total of 5,686 genes clusters were shared among all 4 insects, whereas an additional 1,891 of gene clusters were shared among the 3 species of lice. Notably, *Brueelia* shares a similar number of gene clusters with only *Columbicola* (476) or *Pediculus* (485), even though *Brueelia* and *Columbicola* are in the same family (Philopteridae; although it should be noted that this family is very diverse, and *Brueelia* and *Columbicola* are not closely related, diverging roughly 50 MYA; de Moya *et al*. 2019). Our OrthoVenn analysis identified 41 gene clusters and 501 singletons that are unique to *Brueelia* (Fig. 3b).

### Heterozygosity and demographic history of *B. nebulosa*

**Fig. 4.**
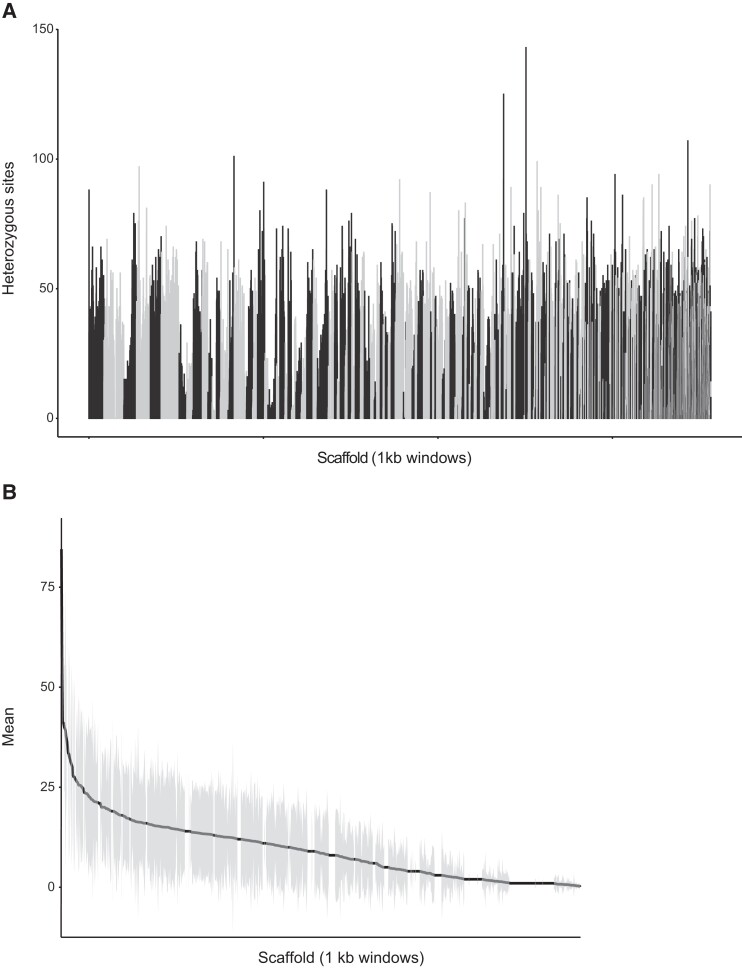
a) Total number of heterozygous sites and b) mean number of heterozygous sites in 1-kbp windows among scaffolds of *Brueelia nebulosa*. Scaffolds in a) are arranged largest to smallest. Scaffolds in b) are arranged from highest mean heterozygosity to lowest; vertical gray lines indicate standard deviation within the 1-kpb windows.

We found 1,006,225 variants (including single nucleotide variants and indels) (0.88%) across the assembled scaffolds, including 956,150 (0.84%) phased variants, 960,026 (0.84%) heterozygous variants, and 748,827 phased heterozygous single nucleotide variants (0.66%) (Supplementary Table 3). These values are smaller than, but consistent with, the estimation of heterozygosity from GenomeScope (1.2%). The distribution of variants was variable among the different scaffolds (Fig. 4). However, scaffolds with the highest numbers of variants (average per 1,000 bp) did not have any annotated genes, suggesting most heterozygosus sites are in noncoding regions. A nearly 1% level of heterozygosity is perhaps surprisingly high for an obligate permanent parasite (Nadler *et al*. 1990; Selman *et al*. 2013). The expectation is that organisms with this lifestyle are more likely to be highly inbred, such as found in seal lice (Virrueta Herrera *et al*. 2022), and/or experience substantial population substructuring (i.e. Wahlund effect), yet the level of heterozygosity suggests otherwise (Plantard *et al*. 2008; 2011). The levels of heterozygosity could also be related to the ecology of the host (European Starling). *Sturnus vulgaris* is a common species and often forms large flocks and roosts (2020). This close contact could facilitate horizontal transmission of their lice and result in a higher genetic diversity than is expected for these parasites. However, genomic data from other lice indicate a similar ∼1% level of heterozygosity, suggesting lice are more mobile than previously assumed. Alternatively, higher heterozygosity could be linked to either mechanisms of chromosomal inheritance biases (e.g. paternal genome elimination; McMeniman and Barker 2006; Gardner and Ross 2014) or elevated mutation rates (Johnson *et al*. 2014), but these hypotheses would require further investigation.

**Fig. 5.**
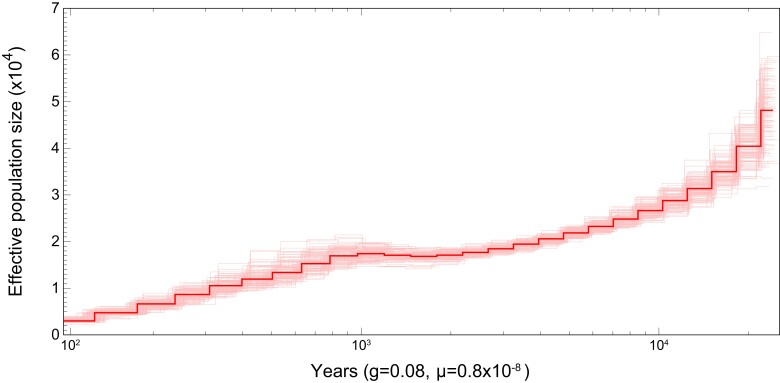
PSMC plot showing estimated effective population size through time in *Brueelia nebulosa*. Solid line shows the estimated values, whereas the lighter lines show results from 100 bootstrap replicates. The time is scaled according to generation time (g; in years) and mutation rate (μ; based on *Drosophila melanogaster*).

Our analysis of ancestral population size indicates a steady decline in N_e_ over the last 1,000 years (Fig. 5). Because the louse was sampled from a North American population of its host, it is possible the decline in effective population size is related to the introduction of several dozen *S. vulgaris* individuals from Europe to North America in the late 19th century. PSMC analyses do not necessarily differentiate between bottlenecks or population structure (Chikhi *et al*. 2010), however, either of these 2 scenarios could be consistent with introduction of the host. Timing of the initial decline in N_e_ does not line up with this hypothesis, but use of a more appropriate mutation rate (we used the rate for *D. melanogaster* in our PSMC analysis) would result in a more reliable date and a stronger test of this scenario. Lice are generally thought to have elevated mutation rates compared to other insects and to their vertebrate hosts (Johnson *et al*. 2014), and using a higher estimate of mutation rate would make the estimate of the bottleneck more recent. PSMC is also known to be less reliable in recovering younger changes (Li and Durbin 2011), so it is possible our estimated decline in N_e_ does indeed reflect the history of intentional introduction of the host.

### Assembly and annotation of the mitochondrial genome

**Fig. 6.**
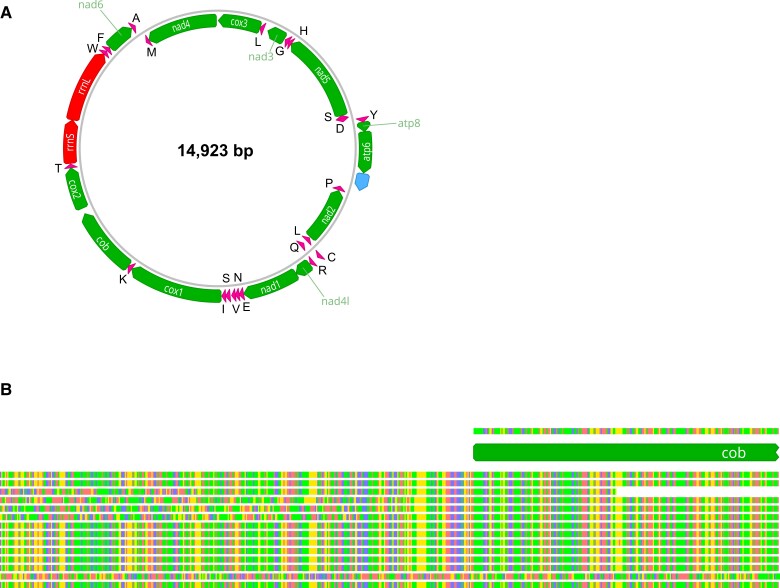
a) Mitochondrial genome (mitogenome) assembled from PacBio reads in *Brueelia nebulosa*. Protein-coding genes are shown in green, transfer RNAs in pink, ribosomal RNAs in red, and the control region in blue. Genes on the light strand are shown on the inside of the circle. b) Example of PacBio reads aligned to a truncated *cob* gene, showing mismatches in bases as evidence for possible heteroplasmy in the mitogenome.

We identified a 14,409 bp contig (mean coverage: 4,206.2) from a BLAST search against *cox1* from *B. antiqua*. The GC content of this contig was 28.7%, which is consistent with other insect mitogenomes (Sweet *et al*. 2020). Our annotation recovered all of the standard 37 mitochondrial genes, including 13 protein-coding genes, 2 ribosomal RNA genes, and 22 transfer RNA genes (Fig. 6a). The arrangement was nearly identical to the mitogenome of *B. antiqua*, the only major differences being indels in nongenic regions (i.e. intergenic or the control region) and the placement of a single tRNA gene (a putative duplication in *B. antiqua*). The overall conservation between the 2 species is notable, given that louse mitogenomes are known to be highly variable in organization and molecular architecture (Shao *et al*. 2009; Cameron *et al*. 2011; Sweet *et al*. 2022). However, our assembled mitogenome is likely incomplete. The *cob* gene, which was at the 3′ end of the assembly, was shorter than expected (564 bp vs > 1,000 bp in other louse mitogenomes). In addition, although AWA indicated a high match (99%), coverage (avg. 4,883.4), and connection coverage (4,680.9) at the 100 bases around the connection between the 5′ and 3′ ends (50 bases on each end), the high alignment scores were not consistently high at all sites (some < −4.0; a good score is > −2.0). It could be there are heteroplasmic arrangements (e.g. a full mitogenome and another smaller fragment containing a subset of genes), which would be challenging for algorithms to assemble de novo. In our assembly, the 5′ end of the *cob* gene consists of repeating thyamines and reads that map to *cob* display considerable variation upstream of the assembled sequence (Fig. 6b). This type of heteroplasmy has been reported with similar patterns (T repeats, alternate read mappings) in other louse taxa (Cameron *et al*. 2011). Because the incomplete mitogenome is likely an artifact of the assembly and heteroplasmy, we took the reads that mapped to the assembled mitogenome in pbmm2 and assembled a subset of these with the native assembler in Geneious. This produced a very long (55,535 bp) contig, but preliminary annotations indicated this was a chimeric assembly of the mitogenome (i.e. repeated multiple times). We then used AWA to identify the complete mitogenome within this long contig and tested for circularity as described above, which strongly supported a complete circle 14,923 bp in length (100% match, alignment score > −0.5 at the connection between 5′ and 3′ ends). Importantly, the annotation in MITOS recovered a *cob* gene within the expected length (1,116 bp), further suggesting we recovered the complete version of the mitogenome.

Finally, a BLAST search against the NCBI nucleotide database recovered a 100% match against a 376 bp portion of *cox1* from a *Brueelia* collected from *S. vulgaris* in Sweden (accession number KT892084), likely *B. nebulosa* (Bush *et al*. 2016). Given the host species and BLAST results, it seems highly likely our specimen is *B. nebulosa*.

### Assembly and annotation of the primary bacterial endosymbiont

**Fig. 7.**
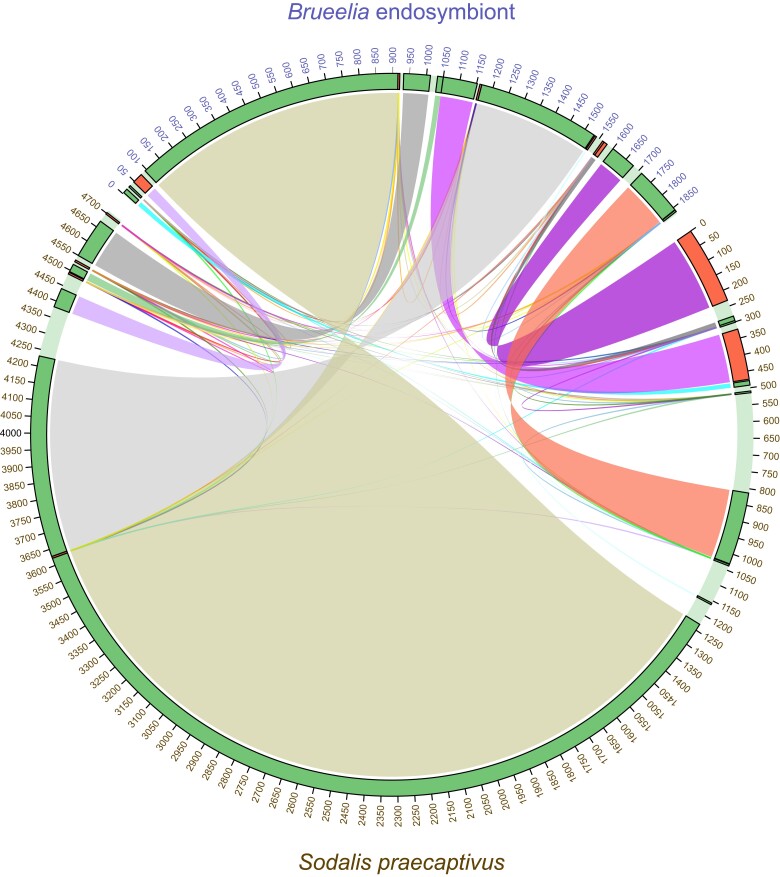
Synteny map of the *Sodalis-*like genome assembled from PacBio reads of *Brueelia nebulosa* compared to the genome (not including the plasmid) of the free-living *Sodalis praecaptivus*. Links are colored according to synteny blocks.

We identified a 1,870,132 bp contig (mean coverage: 168.2, GC%: 53.0%) from BLAST searches against several genes from *Sodalis*. Our AWA analysis suggested the contig is a complete circle, with a 100% match, 248.2 average coverage, 243.0 average connection coverage, and good alignment scores across the connection between the 5′ and 3′ ends of the contig (every site > −1.2). *Sodalis* is the primary endosymbiont in many insects, including some flies, hemipterans, beetles, and lice (Boyd and Reed 2012; Tláskal *et al*. 2021). Our assembled *Sodalis-*like genome is considerably smaller and has a lower GC content than the genome of free-living *S. praecaptivus* (5,159,420 bp, 57.1% GC including the plasmid), but this is expected for endosymbiotic bacteria. Many endosymbionts have reduced genome sizes and lower GC content relative to their free-living relatives, perhaps due to a reliance on the host for certain functions and/or effects of the irreversible accumulation of deleterious mutations (i.e. Müller's Ratchet; Clayton *et al*. 2012; 2014). The size of our assembled *Sodalis*-like genome is larger and has a higher GC content than in other primary endosymbionts from lice, which suggests this lineage of *Sodalis* has not been associated with *Brueelia* for as long as endosymbionts in some other louse taxa. For example, the endosymbiont from *Columbicola wolffhuegeli* is 797,418 bp with 30% GC (Alickovic *et al.* 2021), *Candidatus* Riesia endosymbionts from human lice (582,127 bp, 28.6% GC; Kirkness *et al*. 2010) and chimpanzee lice (576,757 bp with 31.8% GC; Boyd *et al*. 2014). However, our assembled genome is more similar to *Sodalis* endosymbionts in other insects, including in the louse *Proechinophthirus fluctus* from the northern fur seal (*Callorhinus ursinus*; 2,179,576 bp with 50% GC; Boyd *et al*. 2016) and in the carrot psyllid *Bactericera trigonica* (1,575,440 with 55.8% GC; Ghosh *et al*. 2020), suggesting the primary endosymbiont in *Brueelia* has a genome more typical of *Sodalis* endosymbionts. We annotated 2,130 genes or CDS, which is less than half the number of genes in the *S. praecaptivus* genome (4,535). Our synteny analysis indicated large regions in the *S. praecaptivus* genome that are missing in our *Sodalis*-like genome, notably between positions 4.2–4.3 Mb and 500–800 kb (based on NCBI RefSeq NZ_CP006569 for *S. praecaptivus*; Fig. 7). Genes in these regions could be unnecessary for the functioning of an obligate endosymbiont, but future comparative work is needed to more fully understand the functional aspects of any missing genes. At the very least, our *Sodalis*-like genome provides a snapshot into the genomic evolution of bacterial endosymbionts associated with insects.

## Supplementary Material

jkad030_Supplementary_Data

## Data Availability

Reads (HiFi and TELL-Seq) and annotated genome assemblies are available on NCBI under BioProject PRJNA868386. The mitogenome is available under accession GenBank OP353998. Parameters and input files for each analysis are available on figshare: https://doi.org/10.25387/g3.21200377. Supplemental material is available at G3 online.
