# Supplementary figures and images for: Draft genome assemblies of the avian louse Brueelia nebulosa and its associates using long-read sequencing from an individual specimen
Source: G3 (Bethesda). 2023 Feb 3;13(4):jkad030. doi: 10.1093/g3journal/jkad030 (PMC10085802; doi:10.1093/g3journal/jkad030)

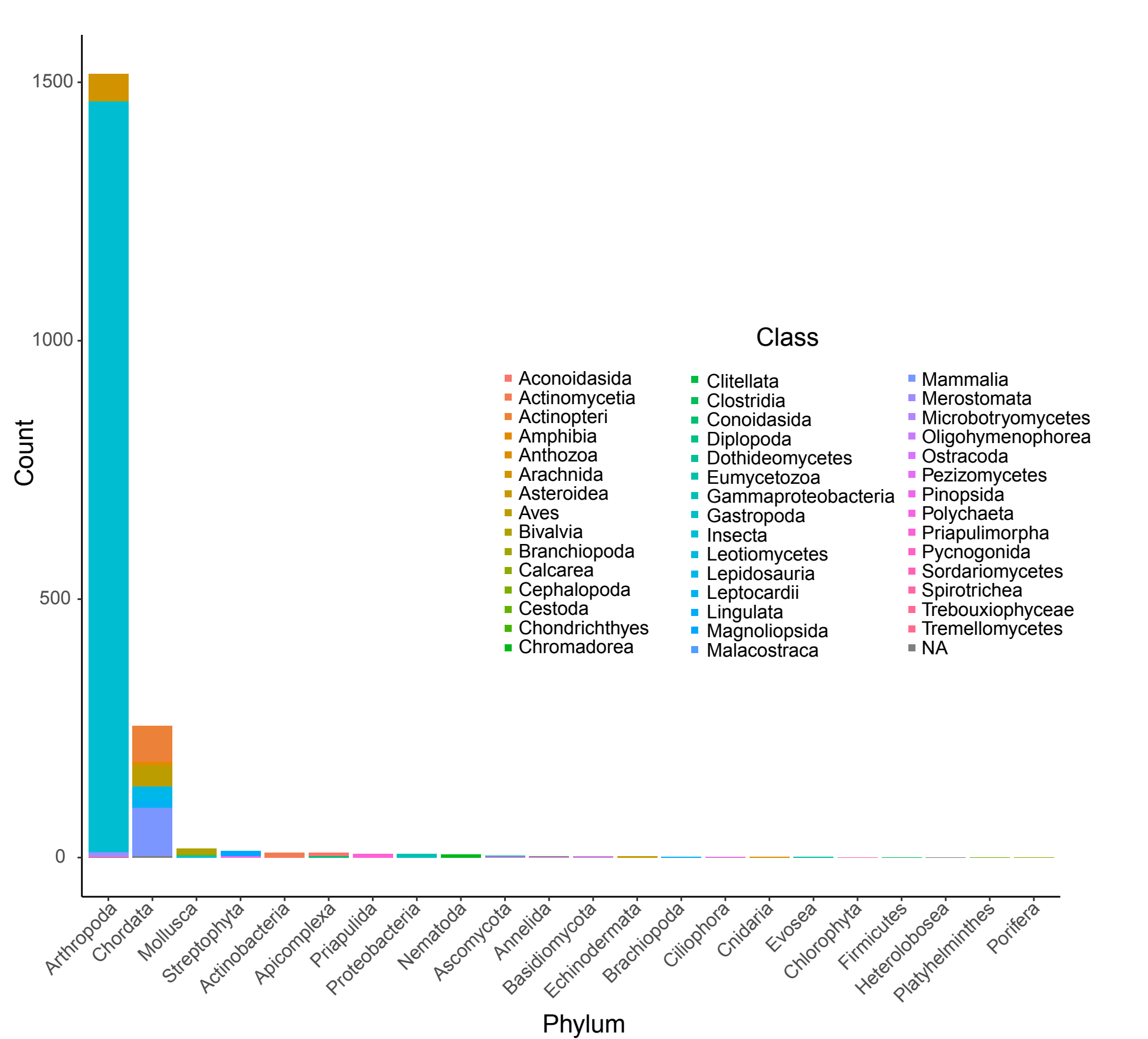

Supplement: jkad030_Supplementary_Data [file jkad030_supplementary_data.zip › Figure_S1_G3-2022-403842.pdf]
